# Dengue diagnosis in Guinea in two returning travelers from Côte d’Ivoire: a case report

**DOI:** 10.1016/j.ijregi.2025.100777

**Published:** 2025-10-03

**Authors:** Jacob Camara, Youssouf Sidibé, Giuditta Annibaldis, Barré Soropogui, Sarah Ryter, Moussa Condé, Eugène Kolie, Ibrahim Nourdine, Bakary Sylla, Carolina van Gelder, Nils Peter Petersen, Mette Hinrichs, Mamadou Diouldé Barry, Soua Koulemou, Bely Sonomy, Mariame Traore, Kaba Keïta, Mamadou Saliou Sow, Mamoudou Conde, Kaba Keïta, Seydou Dia, Moke Fundji Jean Marie Kipela, Fanta Mady Kouyate, Sory Condé, Alimou Camara, Stephan Günther, Sophie Duraffour, Sanaba Boumbaly

**Affiliations:** 1 Centre de Recherche en Virologie - Laboratoire des Fièvres Hémorragiques Virales de Guinée (CRV-LFHVG), Conakry, Guinea; 2 Laboratoire des Fièvres Hémorragiques Virales, Hôpital Régional de N’Zérékoré, N’Zérékoré, Guinea; 3 Bernhard Nocht Institute for Tropical Medicine (BNITM), Hamburg, Germany; 4 German Center for Infection Research (DZIF), partner site Hamburg–Lübeck–Borstel–Riems, Hamburg, Germany; 5 Unité de Maladies infectieuses, Hôpital National de Donka, Conakry, Guinea; 6 Centre de traitement épidémiologique (CTEpi) de N’Zérékoré, N’Zérékoré, Guinea; 7 Hôpital Régional de N’Zérékoré, N’Zérékoré, Guinea; 8 World Health Organization, Conakry, Guinea; 9 Agence Nationale de Sécurité Sanitaire (ANSS), Conakry, Guinea

**Keywords:** Dengue, Diagnosis, West Africa, Guinea

## Abstract

Dengue’s true burden on the African continent is likely underestimated. The majority of cases originate from West Africa, mostly from Burkina Faso. We report here the detection in Guinea of two imported cases of dengue in travelers returning from Côte d'Ivoire, one fatal (case 1) and one mild (case 2). Case 1 lived in Côte d’Ivoire and was diagnosed during a trip in Conakry, Guinea, on 14 August 2023, 9 days after symptom onset. He died on 21 August of an illness related to severe dengue; dengue virus serotype 3 (DENV-3) was further identified. Case 2 was diagnosed in N’Zérékoré, a forested region of Guinea, with serotype 1 (DENV-1) on 11 July 2024, 2 days after disease onset, while returning from Côte d’Ivoire. He was hospitalized for 9 days until full recovery. In-country phylogenetic analysis of case 2 DENV-1 revealed its linkage with urban dengue viruses known to circulate in West Africa. This timely detection, supported by enhanced diagnostics and genomic surveillance capacity in Guinea's laboratory network, highlights the need to strengthen surveillance systems for early detection of emerging viral infections, and emphasizes the importance of further monitoring dengue circulation dynamics in West Africa.

## Highlights

• Detection and identification in Guinea of dengue virus serotype 1 and 3 in returning travelers.
• Strengthened local laboratory capacity enhances Guinea’s disease surveillance.
• Supporting dengue surveillance is crucial for outbreak preparedness in West Africa.
• Research on dengue virus evolution and transmission dynamics in Guinea is needed.

## Introduction

Dengue is a vector-borne viral disease caused by the *Orthoflavivirus denguei* (dengue virus [DENV]), which is transmitted primarily by *Aedes* mosquitoes. The disease poses a significant global public health burden with profound social and economic impacts on populations and health systems [1,2]. Clinical manifestation ranges from mild febrile illness to severe dengue, which can lead to shock and death.

Between 2023 and 2024, a global surge in reported dengue infections was observed, marked by increases in both frequency and magnitude, and emerging even in regions previously unaffected, such as Europe [3]. Key drivers include urbanization, increased vector transmission, population mobility, and the impacts of climate change [1,2]. In the African region, accurately assessing the true burden of dengue remains challenging due to limited surveillance systems, underreporting, and diagnostic constraints [4]. A recent study reported approximately 90,000 confirmed dengue cases and 900 deaths in Africa between 2013 and 2023, with West Africa accounting for 80% of the confirmed cases [4]. All four DENV serotypes (DENV-1, DENV-2, DENV-3, and DENV-4), have been identified in Africa, although DENV-4 detection remains anecdotal [4].

Guinea, a limited-resource country in West Africa, reported only one occurrence of dengue (unknown serotype) in 2006, further confirmed by seroneutralization tests [5]. However, Côte d’Ivoire, Senegal, Mali, Burkina Faso, Ghana, and Benin, among others, have reported increasing sporadic and epidemic circulation of urban and sylvatic DENV-1, DENV-2, and DENV-3 in recent years [4,[6], [7], [8], [9]]. Active dengue circulation in this region is supported by reports of returning travelers diagnosed with DENV [[10], [11], [12], [13]]. Interestingly, modelling studies on dengue importation risk in Africa show a higher risk of importation from within the West African region than from countries outside of Africa [14].

Access to adequate surveillance resources enables timely detection and management of the risks related to dengue’s introduction and establishment. We describe here Guinea’s efficient surveillance and laboratory network response in timely detecting, serotyping, and characterizing two cases of imported dengue in returning travelers in August 2023 (case 1) and July 2024 (case 2). This is the first molecular description of DENV-1 and DENV-3 performed in-country, underlying Guinea’s preparedness programs to enhance public health systems for response to infectious disease outbreaks.

## Case presentation

### Case 1

A 35-year-old Guinean pediatrician (man), living and working in Abidjan, Côte d'Ivoire, left Abidjan on 1 August 2023 and arrived in Conakry, Guinea, on 3 August to attend a work-related event. On 5 August, he reported fever and self-medicated with antimalarial treatment (artemether-lumefantrine, as Bimalaril®) and antipyretic (paracetamol). In the following days, he attended two private clinics and was diagnosed with malaria. On 12 August, he was admitted for severe malaria at the Kobayah clinic in Conakry. One day later, his condition deteriorated with digestive bleeding and vomiting, and he was transferred to the Sino-Guinea Friendship Hospital (HASG, Conakry). The symptoms then included fever, headache, body aches, rhinorrhea, and coffee-ground emesis, which prompted consultation with an infectious disease specialist. Suspicion of dengue and yellow fever led to the sending of an EDTA-blood sample to the reference laboratory in Conakry (*Centre de Recherche en Virologie, Laboratoire des Fièvres Hémorragiques Virales de Guinée*, CRV-LFHVG) on 14 August. Real-time reverse transcription polymerase chain reaction (RT-PCR) was used for dengue and yellow fever virus detection. Viral RNA was extracted (QIAamp viral RNA extraction kit, QIAGEN, Germany), and RT-PCR was performed using the RealStar® Dengue RT-PCR Kit 3.0 and RealStar® Yellow Fever Virus Kit 1.0 (altona Diagnostics GmbH, Germany) on the RotorGene® platform (Qiagen) as in [15]. The sample tested positive for DENV by RT-PCR with a cycle threshold (Ct) value of 31.3. A rapid diagnostic test (RDT), Bioline Dengue immunoglobulin (Ig)G/IgM (Abbott, U.S.), was additionally performed to evaluate the serostatus of the patient. The sample also tested positive for dengue IgG on RDT (IgM negative). Yellow fever RT-PCR was negative. On 14 August, the patient was transferred to the *Centre de Traitement Epidémiologique* (CTEpi) of Nongo, Conakry, for clinical care; he died on 21 August. No other cases were detected. The virus was serotyped as DENV-3 (RealStar® Dengue Type RT-PCR Kit 1.0, altona Diagnostics), and metagenomic nanopore sequencing remained unsuccessful, likely due to low viral load.

### Case 2

**Figure 1:**
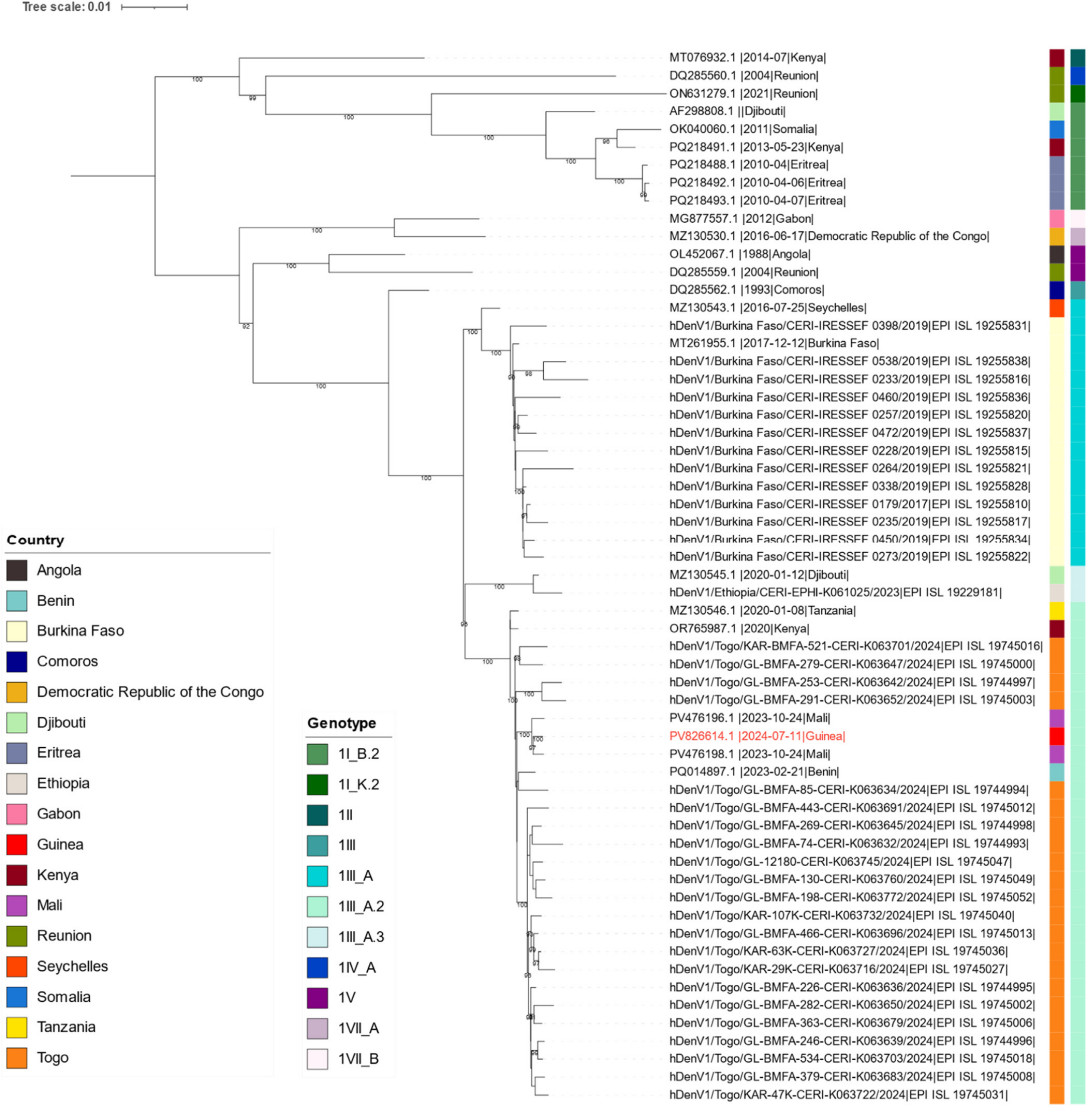
Maximum likelihood phylogenetic tree of case 2. Genomes of dengue virus serotype 1 (DENV-1, n = 58) were selected from NCBI and GISAID repositories to construct the database. Filters included (i) a genome length of at least 9500 base pairs, (ii) geographic location within Africa, (iii) removal of duplicate sequences, and (iv) selection of representative sequences with a similarity of 95% using CD-HIT [17]. Alignment was done with MUSCLE [18]. The tree was drawn using IQ-TREE 2 software for 1000 ultrafast bootstrap replicates under the best model selected by model finder (TIM2+F+I+R3) [19,20]. Visualization was done using the Interactive Tree of Life with bootstrap values ≥90 [21]. Countries and DENV-1 genotypes are depicted as color-coded on the right side of the tree. The complete sequence has been submitted to both GenBank (accession number: PV826614) and GISAID (accession ID: EPI_ISL_20179905). The associated metadata are published in GISAID’s EpiArbo database (EPI_SET_250527db, doi.org/10.55876/gis8.250527db).

A 52-year-old man, living and working in N'Zérékoré, Forest Guinea, traveled by car with three individuals to Côte d'Ivoire from 2 to 8 July 2024, visiting several localities, including Abidjan, Angré, Koumassi, Adjamé, and Plateau. On 9 July, he developed symptoms including fever, asthenia, anorexia, and headaches. Back in N'Zérékoré, he self-medicated with antibiotics (cephalosporin, Ceftriaxone®), a non-opioid analgesic and antipyretic (Metamizole, Novalgin®), antimalarial treatment (Artemether and Lumefantrine, Cofantrine®), and an additional antipyretic (paracetamol, Doliprane®). With persisting asthenia, and following recommendations from his contacts in Côte d’Ivoire—some of whom had recently recovered from dengue—he suspected a dengue infection and sought medical help at the Regional Hospital of N’Zérékoré (HRNZE) on 11 July. Based on dengue suspicion, the laboratory (LFHV-HRNZE) tested the EDTA-blood sample using RT-PCR and dengue RDT (Abbott). The sample was positive for DENV RNA by RT-PCR with a Ct value of 24.4 and for dengue IgM and IgG by RDT. An aliquot was immediately dispatched to CRV-LFHVG, which confirmed the RT-PCR findings with a Ct value of 24.7. The patient then received antibiotics (erythromycin), vitamin C, an antipyretic (paracetamol), and an antimalarial (artemether-lumefantrine, Coartem®). Two follow-up EDTA-blood tests on 16 and 20 July revealed IgG positivity by RDT (IgM negative) and RT-PCR negativity (Ct > 45) on both dates. The patient fully recovered and was discharged on 20 July. No other cases were detected. Molecular serotyping using the RealStar® Dengue Type RT-PCR Kit 1.0 (altona Diagnostics) identified the sample as DENV-1. Metagenomic nanopore next-generation sequencing, basecalling, and consensus generation were performed at CRV-LFHVG as previously described [15]. A 96.7% genome was recovered and further identified as DENV-1 genotype III sub-lineage A.2 (Nextclade Web tool) [16]. Phylogenetic analysis revealed subclustering with recently reported DENV-1 strains from Mali, Benin, and Togo (Figure 1).

DENV, dengue virus.

## Discussion

We report here the in-country detection and molecular characterization of two imported cases of dengue in returning travelers from Côte d’Ivoire: one fatal case identified as DENV-3 (case 1) and one nonfatal case classified as DENV-1 (case 2). Dengue is known to circulate in Côte d’Ivoire, and other reports have previously described similar cases of dengue viruses exported from the country [10,12,13]. Timely testing and diagnosis highlight Guinea’s laboratory readiness and preparedness with the necessary diagnostic tools to survey potentially emerging infections. The laboratory locations, spread across the capital city (CRV-LFHVG) and forest region (LFHV-HRNZE), support the relevance of decentralizing testing facilities close to transport routes or areas where disease outbreaks may emerge [15]. The fatal outcome of case 1 underscores the need for early clinical recognition, improved clinician awareness about dengue, including the relevance of travel history, and enhanced surveillance programs to monitor dengue emergence.

Diagnostic challenges arise from the overlapping symptoms of malaria and dengue in endemic areas, potentially delaying diagnosis and worsening outcomes in co-infected patients [22]. Dengue surveillance in West Africa is fragmented, with limited data on serotype distribution, vector ecology, and mortality rates, highlighting the need for further research [23]. While DENV-1 and DENV-2 are the predominant serotypes in West African outbreaks, evidence linking either to the highest mortality rate remains insufficient [24].

In conclusion, this case report emphasizes the importance of sustained investment in diagnostic capacity and genomic surveillance, and it advocates for expanded regional monitoring, improved clinical awareness, and focused research into the evolution, ecology, and transmission patterns of dengue viruses in Africa.

## Declaration of competing interest

The authors have no competing interests to declare.
